# Amlodipine overdose rescued with extracorporeal membrane oxygenation: A case report

**DOI:** 10.1097/MD.0000000000043534

**Published:** 2025-07-18

**Authors:** Hong Ding, Hui Wang, Zi-Yan Wang, Yuan-Zheng Liu, Kun Zhang

**Affiliations:** a Department of Intensive Care, Affiliated Hospital of Chengde Medical University, Chengde, China; b Department of Intensive Care, Linyi People’s Hospital, Linyi, China.

**Keywords:** amlodipine, calcium channel blocker overdose, extracorporeal membrane oxygenation, hypotension, refractory shock

## Abstract

**Rationale:**

Amlodipine, a calcium channel blocker, is widely prescribed for managing hypertension through vascular smooth muscle relaxation. However, severe amlodipine toxicity can lead to profound hypotension and refractory shock, often unresponsive to conventional treatments.

**Patient concerns:**

In this report, we reported a 32-year-old female case of amlodipine overdose who was admitted to our hospital on March 1, 2024, due to intentionally ingesting approximately 84 tablets of amlodipine (5 mg each).

**Diagnoses:**

Patient was diagnosed with amlodipine overdose and refractory shock.

**Intervention:**

Patient was treated with extracorporeal membrane oxygenation (ECMO), invasive mechanical ventilation, and continuous renal replacement therapy.

**Outcomes:**

On March 7, the patient was successfully weaned off ECMO and discharged on March 15, 2024. During postoperative follow-up, the lung CT scan showed complete resolution of intrapulmonary exudates.

**Lessons:**

The case prompts that early initiation of ECMO, following the failure of conventional medical interventions, was pivotal in stabilizing the patient’s hemodynamics, offering a promising alternative in the management of severe calcium channel blocker toxicity.

## 1. Introduction

Calcium channel blockers (CCBs) are a frequently prescribed class of antihypertensive drugs. They are categorized into 2 main classes based on their chemical structure: dihydropyridines (e.g., nifedipine and amlodipine) and non-dihydropyridines (e.g., verapamil and diltiazem). According to the US Poison Control Center in 2022, CCBs are the 6th most lethal class of exogenous drugs, with a higher mortality rate than that of other antihypertensive agents.^[1]^ Over 5000 cases of CCB overdose are reported per year in the US, with a mortality rate of approximately 50%.^[1]^ Owing to the severity of CCB toxicity, expert recommendations advocate for a multifaceted approach to management, including the administration of vasoactive drugs, rehydration, early intestinal decontamination, calcium and high-dose insulin antagonism, lipid emulsion, and plasma exchange.^[2]^ However, when these measures fail, and patients progress to refractory shock, more advanced life support strategies are required. Extracorporeal membrane oxygenation (ECMO), which provides temporary respiratory and circulatory support, has emerged as a promising intervention in such cases. The current expert consensus recommends the use of veno-arterial ECMO (VA-ECMO) in cases of refractory shock caused by CCB toxicity.^[3]^ This report presents a case of massive amlodipine overdose (420 mg) complicated by refractory shock, which was successfully managed with ECMO.

## 2. Case presentation

**Figure 1.**
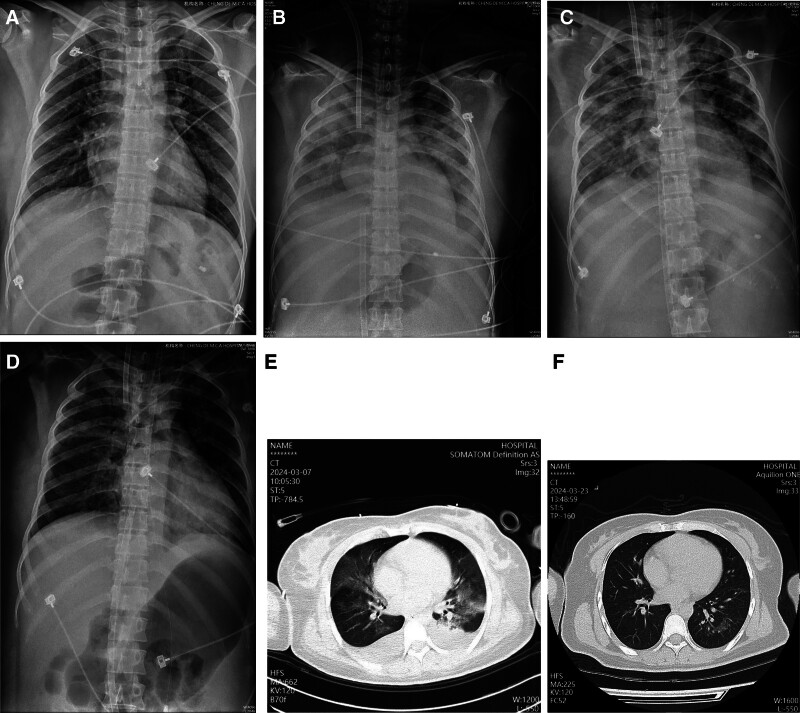
Imaging presentation, transformation, and outcome of the patient. (A) Bedside chest radiography on the day of admission, (B) after 4 hours of VA-ECMO treatment, (C) after VAV-ECMO treatment, (D) bedside chest radiography before ventilator withdrawal, (E) lung CT scan after withdrawal of the ventilator, and (F) lung CT scan before discharge. ECMO = extracorporeal membrane oxygenation, VA-ECMO = veno-arterial ECMO, VAV-ECMO = veno-arterial-venous ECMO.

A 32-year-old female with no medical history intentionally ingested approximately 84 tablets of amlodipine (5 mg each) at 16:00 on March 1, 2024. Her family discovered the overdose approximately 4 hours later and promptly transported her to Weichang County Hospital. On presentation, gastric lavage, rehydration, and other supportive treatments were performed. During this initial treatment, her blood pressure dropped to 80/40 mm Hg, prompting dopamine administration to elevate her blood pressure. However, due to persistent circulatory instability, she was transferred to the intensive care unit (ICU) of the Affiliated Hospital of Chengde Medical College at 21:00 on the same day. Toxicology screening results from the Beijing Gaoxin Hospital revealed 142 ng/mL of amlodipine (normal value < 8 ng/mL). Chest radiography revealed no abnormalities (Fig. 1A). Given the potential for amlodipine toxicity to induce shock, the patient was managed with gastric lavage, 20% mannitol for osmotic catharsis, and a 10% concentrated saline enema for bowel decontamination and detoxification. Additionally, insulin-dextrose infusion (0.5 IU/kg/h), calcium gluconate infusion (30 mL/h), and lipid emulsion infusion (80 mL/h) were initiated to counteract drug toxicity. Continuous renal replacement therapy combined with plasma exchange was performed to remove the accumulated toxins. Five hours later, the patient became drowsy and tachypneic. Blood gas analysis confirmed severe hypoxemic respiratory failure (partial pressure of oxygen [PaO_2_]/fraction of inspired oxygen [FiO_2_] = 100 mm Hg), and the patient was intubated and mechanically ventilated at 02:35 on March 2, 2024.

Despite volume resuscitation and administration of high-dose vasopressors (dopamine, norepinephrine, vasopressin, and epinephrine), her blood pressure remained low at 73/43 mm Hg. Hemodynamic monitoring using the pulse continuous cardiac output indicator system revealed a cardiac output (CO) index of 2.49 L/min/m^2^, systemic vascular resistance index of 1275 dyn·s·cm^‐5^·m^‐2^, global end-diastolic volume index of 525 mL/m^2^, extravascular lung water index of 16 mL/kg, and a central venous pressure of 20 mm Hg. Bedside cardiac ultrasound further revealed reduced myocardial contractility. Serial arterial blood gas analyses showed persistently elevated serum lactate levels exceeding 15 mmol/L, indicating ongoing tissue hypotension and refractory shock secondary to acute amlodipine overdose. Given the lack of improvement, VA-ECMO was initiated as a rescue therapy at 09:30 on March 2, 2024. The pump flow was maintained at 3.5 L/min, sweep gas flow at 5 L/min, and inspiratory oxygen concentration (FiO_2_) at 1.0. Within 6 hours, the patient’s blood pressure gradually increased from 60/42 mm Hg to 145/65 mm Hg, allowing for a reduction in vasoactive drugs. Ultrasound showed improvement in cardiac systolic function [left ventricular ejection fraction (LVEF) = 60%, CO = 4.36 L/min], and her hemodynamics gradually stabilized. Despite ongoing ECMO support, the patient developed refractory hypoxemia. Arterial blood gas analysis revealed a marked oxygenation gradient: the PaO_2_ in the right radial artery was low at 28 mm Hg, while that in the right femoral artery was elevated at 489 mm Hg, despite 100% FiO_2_ and a positive end-expiratory pressure (PEEP) setting of 14 cm H_2_O. Chest radiography confirmed worsening pulmonary edema (Fig. 1B). Physical examination revealed differential cyanosis, characterized by dusky discoloration of the upper body and normal skin tone in the lower extremities (clinical features consistent with North-South syndrome). Veno-venous ECMO (VV-ECMO) was subsequently initiated to address refractory hypoxia. However, within 3 hours of VV-ECMO, the patient’s hemodynamic status deteriorated, with blood pressure decreasing to 68/45 mm Hg and norepinephrine infusion escalating to 5 μg·kg^‐1^·min^‐1^. Ventilator settings remained high (tidal volume of 420 mL/kg, pressure support of 14 cm H_2_O, PEEP of 14 cm H_2_O, respiratory rate of 18 breaths/min, and FiO_2_ of 100%). Oxygen saturation remained unstable, fluctuating between 85% and 95%. Repeat arterial blood gas analysis of the right radial artery showed that pH was 7.51, PaO_2_ was 98 mm Hg, PaCO_2_ was 22 mm Hg, and lactate was 9.6 mmol/L. A repeat bedside cardiac ultrasound revealed a reduction in LVEF (LVEF = 50%, CO = 2.50 L/min), suggesting further deterioration in cardiac function. Given the persistent respiratory and circulatory failure, the decision was made to implement veno-arterial-venous ECMO (VAV-ECMO). VAV-ECMO was initiated *via* a left femoral vein, with blood divided through an oxygenator. The blood flow was adjusted using a Hoffman clamp: one portion was returned to the right internal jugular vein to improve the oxygenation of the upper limbs, and the remainder returned to the right femoral artery to support systemic circulation. ECMO parameters were set to a pump flow of 5.2 L/min, sweep gas flow of 5 L/min, and FIO_2_ at 1.0, with femoral arterial perfusion at 2.2 L/min and internal jugular vein perfusion at 2.4 L/min.

After 12 hours of VAV-ECMO, the patient demonstrated significant hemodynamic improvement. The norepinephrine infusion was reduced to 0.5 μg·kg^‐1^·min^-1^, and her blood pressure stabilized at 128/67 mm Hg. In parallel, ventilator parameters were gradually de-escalated, with adjustments to a tidal volume of 420 mL, pressure support of 14 cmH_2_O, PEEP of 12 cm H_2_O, and FiO_2_ of 50%. Arterial blood gas analysis of the right radial artery showed a PaO_2_ of 98 mm Hg and a lactate level of 2.3 mmol/L, confirming the stabilization of circulatory function. Echocardiography confirmed improved cardiac contractility, while chest radiography revealed exudative shadows in both lungs (centered at the hilum) (Fig. 1C), indicative of severe pulmonary edema. After a complete evaluation, the patient was transitioned back to VV-ECMO at 10:00 on March 3, 2024. As ECMO support continued, norepinephrine was gradually discontinued, and a negative fluid balance was achieved.

**Figure 2.**
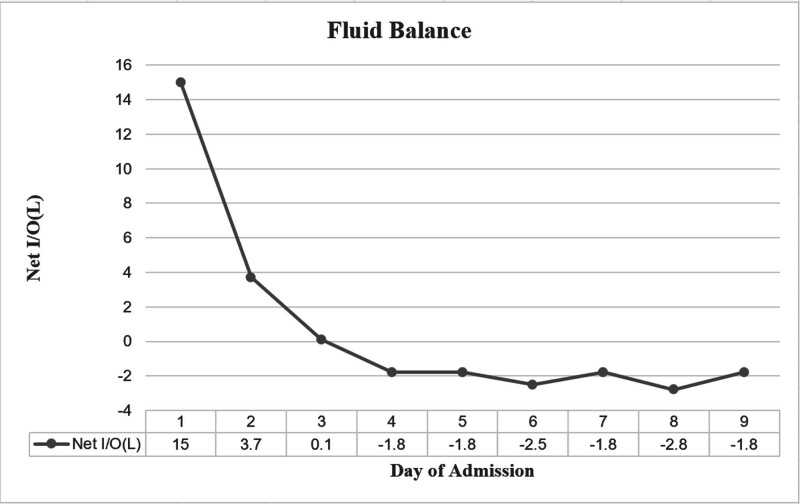
Fluid balance.

**Figure 3.**
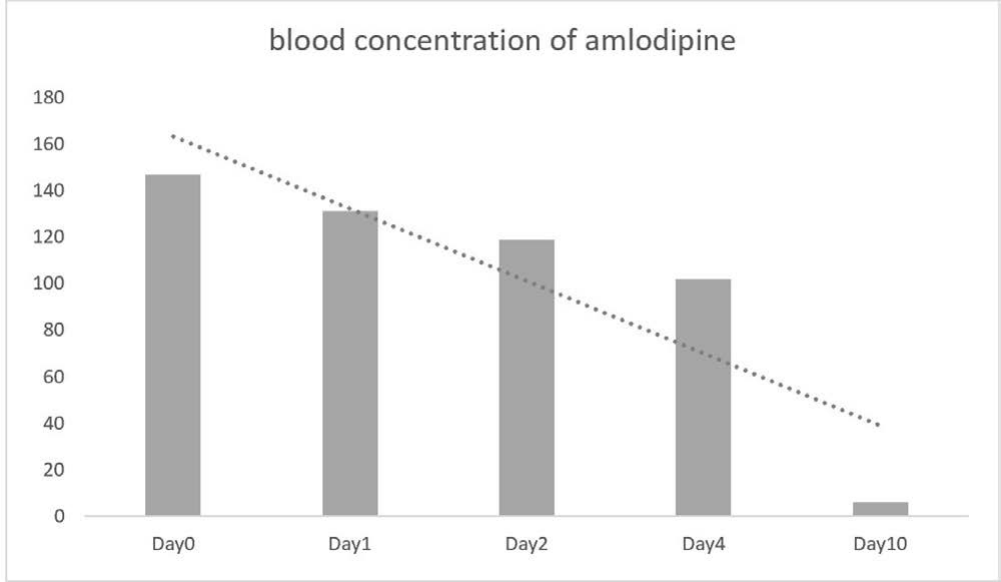
Blood concentration of amlodipine.

**Figure 4.**
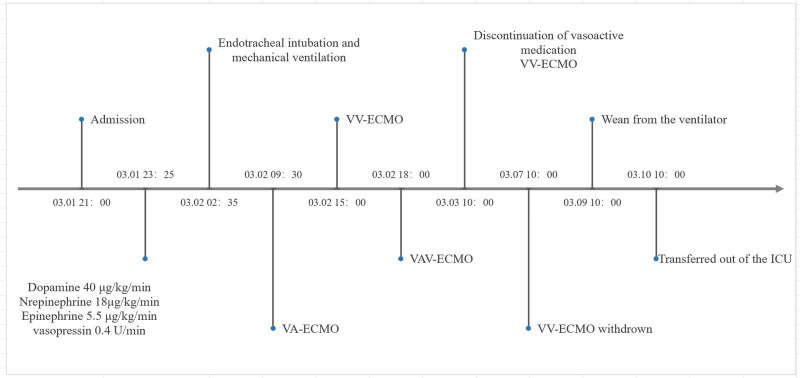
Timeline of the patient’s disease course and management.

On the 5th day in the ICU, chest radiography showed significant improvement in pulmonary edema (Fig. 1D). The patient was successfully decannulated within 120 hours post-ECMO. On day 7, a lung CT scan revealed mild residual exudates within the lungs (Fig. 1E). The ventilator parameters were gradually adjusted downward, and the patient was successfully extubated. By day 10, the patient was discharged from the ICU, with a follow-up lung CT scan showing complete resolution of intrapulmonary exudates (Fig. 1F). Figure 2 illustrates the patient’s fluid balance throughout the course of treatment. Toxicology screening confirmed a gradual decline in amlodipine concentration to 6 ng/mL (Fig. 3). After a 3-month follow-up, the patient remained in excellent health. A detailed timeline of the patient’s clinical course and management is shown in Fig. 4.

## 3. Discussion

Amlodipine belongs to the dihydropyridine group of the CCBs. It can reduce peripheral vascular resistance by acting on peripheral vascular L-calcium channels, thereby causing arteriolar vasodilation.^[4]^ However, amlodipine overdose can cause severe hypotension, intractable shock, and reflex tachycardia. Initial management of patients with amlodipine toxicity focuses on stabilizing the circulatory system, typically through fluid infusion and vasopressor medications. In such cases, norepinephrine is the preferred vasoconstrictor; however, when it proves ineffective, a combination with epinephrine or terlipressin can be considered.^[5]^ Recent studies have shown that methylene blue can impede peripheral vasodilation by inhibiting guanylate cyclase and NO synthase.^[6]^ When the elevation in blood pressure is suboptimal, methylene blue can be administered in combination. If blood pressure remains refractory to these treatments, advanced life support should be planned as expeditiously as possible.

In this case, the acute pulmonary edema that developed during treatment was considered to be non-cardiogenic pulmonary edema induced by amlodipine toxicity with a multifactorial etiology. First, amlodipine selectively dilates precapillary resistance vessels, resulting in augmented capillary hydrostatic pressure and interstitial pulmonary edema.^[7]^ Second, aggressive fluid resuscitation can enhance pulmonary capillary permeability in cases of refractory shock, thereby exacerbating pulmonary edema.^[8]^ Third, CCBs inhibit the secretion of surface-active substances by alveolar type II epithelial cells, resulting in alveolar collapse.^[9]^ In this case, aggressive fluid resuscitation and maintenance with high doses of vasopressor medications failed to stabilize circulation. Therefore, rapid and massive administration of crystalloids may be a risk factor for pulmonary edema. While early stabilization of circulation is essential, vigilance for non-cardiogenic pulmonary edema during fluid resuscitation remains necessary.

This report presents a case of severe amlodipine toxicity successfully managed using a VAV-ECMO model. The current expert consensus recommends that VA-ECMO be used in cases of refractory shock caused by CCB toxicity.^[3]^ In this case, the VA-ECMO model was initially used because of the severe hypotension and refractory hypoxemia. Following the development of North-South syndrome, the VV-ECMO model was chosen. Finally, the patient was transitioned to a VAV-ECMO model after the onset of circulatory collapse. Weinberg et al reported the use of VA-ECMO (8 days) in patients experiencing shock and respiratory failure due to an overdose of amlodipine, lysergic acid, or hydrochlorothiazide.^[10]^ Stycuła et al concluded that VV-ECMO is a prudent choice without significantly compromising cardiac performance.^[11]^ This is attributable to the working principle of VV-ECMO, which provides oxygenation support while reducing ventilator-associated lung injury, thereby preserving cardiac function. Additionally, VV-ECMO can prevent the initiation of the North-South syndrome, which is a complication associated with VA-ECMO. Haughey et al reported a case of amlodipine toxicity in a patient with normal left ventricular function, where VV-ECMO was successfully used to manage refractory hypoxemia and achieve full recovery.^[12]^ This case highlights the utility of VV-ECMO in amlodipine-intoxicated patients who maintain normal left ventricular function but experience refractory failure. Conversely, in patients with refractory shock secondary to reduced myocardial contractility (an established complication of amlodipine toxicity) early initiation of VA-ECMO is recommended to restore hemodynamic stability. Nevertheless, clinicians should remain alert for the emergence of North-South syndrome. Prompt recognition and adjustment of the ECMO configuration are essential to prevent cerebral and myocardial hypoxia. For patients with refractory shock and refractory hypoxemia despite conventional treatments, such as aggressive fluid resuscitation, circulatory support, and adjustment of ventilator parameters, VAV-ECMO may be a comprehensive support modality. Therefore, in patients with severe CCB toxicity who are hemodynamically unstable and refractory to conventional treatment, ECMO should be initiated as early as possible to provide prompt, temporary support for both the respiratory and cardiovascular systems, allowing time for toxicant metabolism and recovery of organ function. Due to cardiac dysfunction, most documented cases of amlodipine toxicity are now managed using the VA-ECMO model. In contrast, the use of VV-ECMO and VAV-ECMO models in amlodipine-related cases remains relatively uncommon, underscoring the need for further clinical experience to guide their application. In the treatment of distributive shock induced by amlodipine toxicity, dynamic assessment of intravascular volume status is essential, particularly during aggressive fluid resuscitation vasopressor titration. Excessive fluid administration may exacerbate capillary leakage and lead to noncardiogenic pulmonary edema, increasing the risk of refractory hypoxemia.

## 4. Conclusion

Severe amlodipine toxicity can lead to vasodilatory shock. In cases of ineffective conventional treatment, the early implementation of ECMO for the stabilization of hemodynamic parameters represents a novel therapeutic approach for patients with CCB overdose. Patients with CCB toxicity can develop multiple life-threatening complications. Therefore, the prompt modification of treatment protocols may be instrumental in reducing patient mortality. Notably, fluid resuscitation may result in a sudden increase in volume. Thus, ongoing assessment and management of acute pulmonary edema induced by amlodipine overdose is necessary.

## Acknowledgments

We acknowledge TopEdit LLC for the linguistic editing and proofreading during the preparation of this manuscript.

## Author contributions

**Conceptualization:** Hong Ding, Hui Wang, Zi-Yan Wang.

**Data curation:** Hong Ding, Zi-Yan Wang.

**Investigation:** Hui Wang.

**Methodology:** Zi-Yan Wang.

**Writing – original draft:** Hong Ding, Hui Wang.

**Writing – review & editing:** Yuan-Zheng Liu, Kun Zhang.
